# Special Issue on “Infrared Thermography and Additional Non-Destructive Testing for Building, Structure and Material Inspections”

**DOI:** 10.3390/s21093107

**Published:** 2021-04-29

**Authors:** Stefano Sfarra, Eva Barreira, Susana Lagüela

**Affiliations:** 1 Department of Industrial and Information Engineering and Economics, University of L’Aquila, Piazzale E. Pontieri n. 1, 67100 L’Aquila, Italy; 2 CONSTRUCT-LFC, Faculty of Engineering (FEUP), University of Porto, Rua Dr. Roberto Frias s/n, 4200-465 Porto, Portugal; barreira@fe.up.pt; 3 Cartographic and Land Engineering Department, Higher Polytechnic School of Avila, University of Salamanca, Hornos Caleros, 50 05003 Avila, Spain; sulaguela@usal.es

Non-destructive testing (NDT) describes techniques that measure properties of the body without disturbing their state. Infrared thermography (IRT) is a special type of NDT that evaluates the thermal state of the bodies, enabling the determination of the presence of thermal pathologies. It can be applied autonomously or combined with other NDT techniques that provide additional information to complete the study of a particular element, for example, to provide information about the interior of an element (higher depth). Thus, different IRT methods, alone or together with other NDT techniques, stand as good alternatives for the evaluation of the state of structures and building materials that cannot be “touched” for several reasons [1,2,3].

This Special Issue of *Sensors* “Infrared Thermography and Additional Non-Destructive Testing for Building, Structure and Material Inspections” revolves around both review and original research manuscripts related to the application of IRT and other non-destructive testing techniques to the inspection of buildings, infrastructures and materials for the detection, identification and characterization (geometric and thermal) of pathologies that affect the integrity of the element under study.

Through smart scientific approaches oriented towards the analysis of thermal and mechanical responses, this Special Issue will help readers and future generations of NDT technicians to become more familiar with knowledge frontiers and foster an increased interest in the field.

The guest editors thought to collect the latest ideas on relevant topics, and more importantly, to address present challenging issues with the use of IRT possibly applied in combination with other NDT techniques on different targets. There were 10 manuscripts collected in this Special Issue. Various research topics have been addressed, mainly (but not limited to) temperature-dependent and/or mechanical-dependent phenomena related to the engineering, physics, artistic and architectural fields.

In particular, Barreira et al. presented an innovative contribution, both to the characterization of the emissivity of various construction materials, and to the discussion of emissivity measurement procedures and attendant uncertainty. The results confirmed that emissivity is a crucial parameter for the accurate measurement of surface temperature. Emissivity measurements carried out with IRT (black tape method) and with an emissometer returned meaningful differences when compared with the values available in the literature [4].

Lerma et al. compared three approaches on a real case study, such as monitoring with temperature and relative humidity sensors, finite element simulations—computational fluid dynamics (CFD) and thermographic images (IRT). The work provided an assessment of the risk of condensation over a year and identified periods and areas of the building affected by potential problems. Sensors and IRT pictures provided real data to validate CFD simulations, facilitating a global analysis of the building. The results provided reflect a great concordance between the NDT techniques used [5].

In the work of Wei et al., artificial intelligence was applied in combination with IRT to detect and segment the impact damage on curved laminates that were previously submitted to severe thermal stress cycles and subsequent ballistic impacts. Segmentation was performed on both mid-wave and long-wave infrared sequences, obtained simultaneously during pulsed thermography experiments, by means of a deep neural network. A deep neural network was trained for each wavelength. Both networks generated satisfactory results [6].

In [7], Hu et al. presented an enhanced framework using sparse pattern extraction in the processing of a thermographic sequence to detect defects. This framework adapted cropping operator and typical component extraction as a preprocessing step to reduce the dimensions of raw data and applied sparse pattern extraction algorithms to enhance the contrast on the defective area. Different cases were studied, involving several defects in four basalt–carbon hybrid fiber-reinforced polymer composite laminates. Finally, comparative analyses with intensity distribution were carried out to verify the effectiveness of contrast enhancement using this framework.

The manuscript by Garrido et al. introduced the joint application of IRT and ground-penetrating radar (GPR) for the detection and classification of the moisture in the interior walls of a building according to its severity level. The complementarity of both methods has proven to be an effective approach to investigate both surface and internal moisture and their severity. Specifically, IRT allowed estimating superficial water movement, whereas GPR allowed detecting points of internal water accumulation. Thus, through the combination of both techniques, it was possible to provide an interpretation of the water displacement, from the exterior surface to the interior surface of the wall, and to provide a relative depth of water inside the wall. It was concluded that more information and greater reliability can be gained by using complementary IRT-GPR, showing the benefits of combining both techniques in the building sector [8].

Garrido et al. also performed a study on a tessellatum layer of a mosaic to automatically: (i) detect the first appearance of the thermal footprint of internal water; (ii) delimit the contours of the thermal footprint of internal water from its first appearance; and (iii) distinguish between harmful and non-harmful internal water. The study was based on the analysis of the temperature distribution of each thermal image. Five thermal image sequences were acquired during the simulation of different real situations, obtaining a set of promising results for the optimization of the thermographic inspection process, while discussing the following recommended steps to be taken in future research [9].

The relevance of the manuscript of Barreira et al. [10] was related to the discussion of the strengths and weaknesses of several methods to quantitatively assess the humidification phenomenon using IRT. For that purpose, the partial humidification via the bottom surface of a lightweight concrete specimen was considered as a case study. To evaluate the thermal gradients, the evolution of the thermal imaging throughout the measurement period and the definition of the areas particularly affected by moisture, a methodology that included a pre-processing phase for data reduction, followed by a data processing phase, were implemented. In data processing, different statistical and numerical methods were tested. The results of the statistical descriptive analysis highlighted the time variation of the surface temperature, both when considering the entire specimen and when considering only specific areas. The variability of the temperatures at certain moments of the experiment was observed and discussed. The image subtraction proved to be an interesting technique to quantify the temperature differences if the first image was used as reference. A thermal index, TI, was proposed to assess the cooling rate. The index highlighted the initial instant when the effect of moisture on the surface temperature was detectable.

An integrated study of two NDT techniques applied on wooden samples is presented in [11]. IRT was able to retrieve thermal parameters of the wood related to the amount of water added to the samples, while the interference pattern generated by speckles was used to quantify the expansion and contraction of the wood, which was related to the amount of water. Twenty-seven wooded samples were used, in which a known quantity of water was added in a controlled manner. By applying advanced image processing to thermograms and specklegrams, it was possible to determine the fundamental values controlling both the absorption of water and the main thermophysical parameters that linked the samples. On the one hand, the proposed results should be considered preliminary because the experimental values obtained by IRT need to be optimized for low water contents; on the other hand, speckle interferometry—by applying an innovative procedure—provided robust results for both high and low water contents.

In [12], IRT and hyperspectral imaging methods were applied to identify both fabricated and non-fabricated targets in a canvas painting, alluding to the famous character “Venus” by Botticelli. The pulse-compression thermography technique was used to retrieve information concerning the inner structure of the sample, and low power light-emitting diode (LED) chips, of which the emission was modulated via a pseudonoise sequence, were exploited as the heat source for minimizing the heat radiated on the sample surface. Hyper-spectral imaging was employed to detect surface and subsurface features such as pentimenti and facial contours. The results demonstrated how the application of statistical algorithms (i.e., principal component and independent component analyses) maximized the number of targets retrieved during the post-acquisition steps for both employed techniques. Finally, the best results obtained by both techniques and post-processing methods were fused together, resulting in a clear targets map, in which the surface, subsurface and deeper information were all shown at a glance.

Lastly, in [13], two different NDT techniques, namely active IRT and ultrasonic testing, were used to detect a delamination in a glass/silicone composite. A frequency-modulated chirp signal and pulse-compression were proved to successfully be used in active IRT for detecting such delamination. Moreover, the same type of input signal and post-processing was used to generate an image using air-coupled ultrasound, and an interesting comparison between the two was made to further characterize the defect.

In summary, there is a huge potential for IRT and additional NDT techniques for monitoring and sensing buildings, structures and materials. However, there are still many challenges to address before those techniques can become a reliable reality in each sector involving diagnostics.

It is possible to say that many researchers in the past underestimated those challenges. They were highly optimistic, based solely on oversimplified modelling, simulations or quick lab tests, only to be disappointed later by the outcome of in-depth verifications, mainly in situ.

The potential of IRT and additional NDT techniques is great, but the challenges are great too. Nevertheless, we all know that great challenges generate great, rewarding, and impactful research—without challenges, research is boring and meaningless.

The present Special Issue selected serious works based on several, deep tests, confirming the first findings. The Guest Editors leave the doors open for future ideas concerning Special Issues centered on conscientious research activities, aimed at improving the future of NDT techniques.

## Data Availability

Not applicable.
